# Using an Interactive App for Symptom Reporting and Management Following Pancreatic Cancer Surgery to Facilitate Person-Centered Care: Descriptive Study

**DOI:** 10.2196/17855

**Published:** 2020-06-17

**Authors:** Tina Gustavell, Kay Sundberg, Ann Langius-Eklöf

**Affiliations:** 1 Karolinska Institutet Department of Neurobiology, Care Sciences and Society Division of Nursing Stockholm Sweden; 2 Karolinska University Hospital Theme Cancer Department of Upper Abdominal Diseases Stockholm Sweden

**Keywords:** pancreatic neoplasms, mHealth, interactive, symptoms, self-care, support, patient-reported outcomes, person-centered care

## Abstract

**Background:**

Pancreatic and periampullary cancers are rare but have high mortality rates. The only hope for cure is surgical removal of the tumor. Following pancreatic surgery, the patients have a great deal of responsibility for managing their symptoms. Patients report a lack of sufficient knowledge of self-care and unmet supportive care needs. This necessitates a health care system responsive to these needs and health care professionals who pay close attention to symptoms. Person-centered care is widely encouraged and means a shift from a model in which the patient is the passive object of care to a model involving the patient as an active participant in their own care. To address the challenges in care following pancreatic cancer surgery, an interactive app (Interaktor) was developed in which patients regularly report symptoms and receive support for self-care. The app has been shown to reduce patients’ symptom burden and to increase their self-care activity levels following pancreaticoduodenectomy due to cancer.

**Objective:**

The aim of the study was to describe how patients used the Interaktor app following pancreaticoduodenectomy due to cancer and their experience with doing so.

**Methods:**

A total of 115 patients were invited to use Interaktor for 6 months following pancreaticoduodenectomy. Of those, 35 declined, 8 dropped out, and 46 did not meet the inclusion criteria after surgery, leaving 26 patients for inclusion in the analysis. The patients were instructed to report symptoms daily through the app for up to 6 months following surgery. In case of alerting symptoms, they were contacted by their nurse. Data on reported symptoms, alerts, and viewed self-care advice were logged and analyzed with descriptive statistics. Also, the patients were interviewed about their experiences, and the data were analyzed using thematic analysis.

**Results:**

The patients’ median adherence to symptom reporting was 82%. Fatigue and pain were the most reported symptoms. Alerting symptoms were reported by 24 patients, and the most common alert was fever. There were variations in how many times the patients viewed the self-care advice (range 3-181 times). The most commonly viewed advice concerned pancreatic enzyme supplements. Through the interviews, the overarching theme was “Being seen as a person,” with the following 3 sub-themes: “Getting your voice heard,” “Having access to an extended arm of health care,” and “Learning about own health.”

**Conclusions:**

Interaktor proved to be well accepted. It made patients feel reassured at home and offered support for self-care. The app facilitated person-centered care by its multiple features targeting individual supportive care needs and enabled participation in their own care. This supports our recent studies showing that patients using the app had less symptom burden and higher self-care activity levels than patients receiving only standard care.

## Introduction

Pancreatic and periampullary cancers are rare, with only 1300 individuals (equal proportions of men and women) diagnosed each year in Sweden [1]. The mortality rate is high because surgical resection can only be offered to fewer than 20% of patients [2]. Even after intentionally curative surgery and adjuvant chemotherapy, the prognosis is poor, with a median survival period of 2-4 years depending on whether it is pancreatic or periampullary cancer [3,4]. The most common surgical procedure for these tumors is pancreaticoduodenectomy, which impairs quality of life [5,6]. High demands are put on patients to manage their illness after surgery. It has been concluded that patients who have had a pancreaticoduodenectomy sometimes lack sufficient knowledge of self-care and have unmet supportive care needs, which necessitate a health care system that is responsive to these needs and health care professionals who pay close attention to symptoms [7,8].

Patients must often navigate through a fragmented health care system and adapt to routines customized to the health care organizations and professionals, rather than receiving care designed to focus on the individual patient’s needs, preferences, and values [9]. Person-centered care is today a widely encouraged alternative and means a shift away from a model in which the patient is the passive object of care to a model where arrangements are made involving the patient as an active participant in his or her care [10]. Participation in one’s own care can include mutual communication with health care professionals where patients are listened to and their knowledge is respected, shared knowledge where patients receive explanations of symptoms and procedures and can also tell professionals about their symptoms, and patients knowing how to manage their symptoms and provide self-care [11]. To achieve person-centered care where patients really are active participants, support of a positive attitude to modern innovations is needed. Routine use of patient-reported outcomes in clinical practice can be one way of identifying patients’ current concerns and impact of treatment, enhancing patient-clinician communication, promoting shared decision making, and improving patient satisfaction [12,13]. Medical and public health practices supported by mobile devices have been defined by the World Health Organization as mobile health (mHealth) [14]. It has been reported that patients undergoing cancer treatments who report symptoms to health care professionals through mHealth systems and receive support for symptom management have higher quality of life, less symptom distress [15-17], and improved 2-year survival [18] compared with patients not using such systems.

Given the poor prognosis of pancreatic and periampullary cancer, the distressing symptoms patients experience, and insufficient knowledge of self-care and unmet supportive care needs, challenges arise in supporting patients with cancer following pancreaticoduodenectomy. To address these challenges, an interactive app (Interaktor) for smart devices was developed in which patients regularly report symptoms and receive support through continuous access to self-care advice and their health care professionals. The content in the app was developed by reviewing literature and interviewing patients and health care professionals [19] and has been tested for feasibility [20]. Evaluation of the app’s impact on quality of life has shown higher emotional function and less symptom burden 6 weeks after surgery for patients using the app compared with patients not using the app [21]. Furthermore, patients using the app had higher self-care activity levels 6 months after surgery [21]. Knowledge of the patients’ usage and experience of the app may support the interpretation of these results. Therefore, the aim of the current study was to describe how patients used the Interaktor app following pancreaticoduodenectomy due to cancer and their experience with doing so.

## Methods

### Design

The current study is part of the evaluation of the Interaktor app adjusted for patients with pancreatic cancer and has a descriptive design. Ethical approval was given by the Regional Ethical Review Board in Stockholm, Sweden (Reg.no: 2011/1780-13/2).

### Setting

The study was performed at Karolinska University Hospital, which has the highest volumes of pancreatic surgery in Sweden. Following pancreaticoduodenectomy, at the time of the study, the patients were normally cared for on a surgical ward for 1 to 2 weeks and thereafter at a rehabilitation unit outside the hospital for 1 week. Standard care after discharge was that the patients should contact the clinic’s outpatient unit if they felt the need to. Also, around 5 weeks after surgery, the patients had an appointment with a surgeon at the outpatient unit. After this appointment, patients with a confirmed diagnosis of malignant disease were referred to the oncology clinic to start adjuvant chemotherapy. The chemotherapy had to start within 10 weeks after surgery, and standard treatment was gemcitabine given as an intravenous infusion over 30 minutes, once a week for 3 of every 4 weeks (1 cycle), for 6 cycles.

### Sample

**Figure 1:**
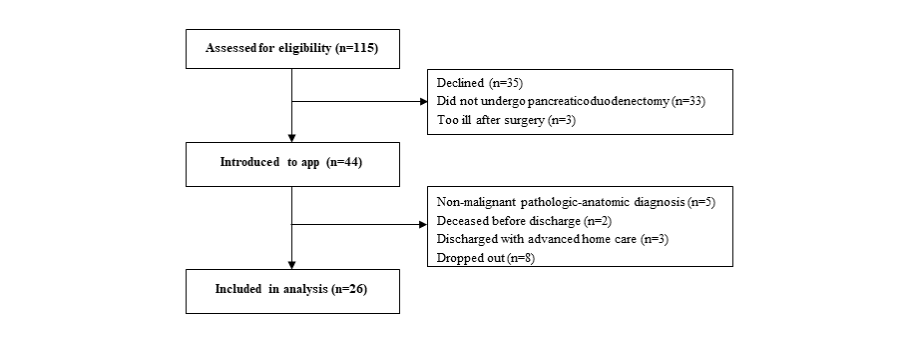
Flowchart of the inclusion process.

During a period of 16 months in 2015-2016, all patients who were scheduled to undergo pancreaticoduodenectomy at the university hospital due to a suspected malignancy in the pancreatic or periampullary region were screened for eligibility. Inclusion criteria were follow-up care planned at the university hospital and able to read and understand Swedish. After the screening process, 115 patients were eligible before surgery. A total of 35 patients declined to participate. After surgery, patients who did not undergo pancreaticoduodenectomy or were too ill were excluded. Upon discharge, 44 patients were introduced to the app. Patients who did not have malignant disease, who died before discharge, who were discharged with advanced home care, or who dropped out were not analyzed, leaving a final sample of 26 patients included in the analysis (Figure 1). Characteristics of participants included in the analysis are shown in Table 1.

**Table 1:** Sociodemographic and clinical characteristics of the study participants (n=26).

Sociodemographic and clinical characteristics			Descriptive analyses, n (%)
Age (years)			67 (8.7)^a^, 67 (51-82)^b^
**Sex**			
	Female	8 (31)	
	Male	18 (69)	
**Living situation**			
	Married or living with partner	21 (81)	
	Living alone	5 (19)	
**Highest education level**			
	Junior compulsory	1 (4)	
	Senior high school	9 (35)	
	Postgraduate or university	15 (58)	
	Missing data	1 (4)	
**Histopathology**			
	Pancreatic ductal adenocarcinoma	12 (46)	
	Periampullary cancer	12 (46)	
	Invasive intraductal papillary mucinous neoplasia	2 (8)	
**Adjuvant chemotherapy**			
	Yes, full cycle	17 (65)	
	Yes, ceased in advance^c^	5 (19)	
	No	4 (15)	

^a^mean (SD).

^b^median (range).

^c^Due to side effects (n=2), recurrent disease (n=2), or death (n=1).

### Interaktor

**Figure 2:**
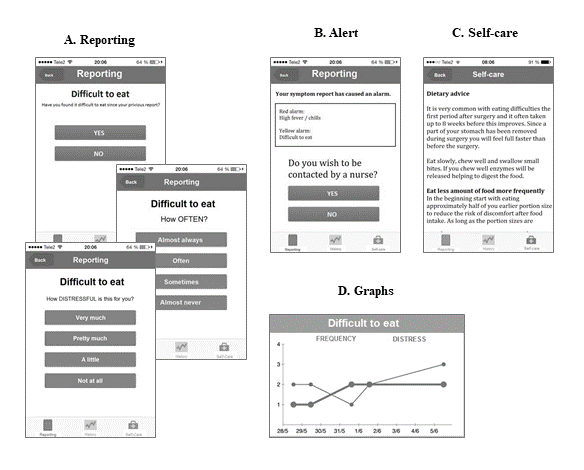
Screenshots from the Interaktor app adapted for patients following pancreaticoduodenectomy showing the primary features: (A) symptom reporting, (B) alerts, (C) self-care advice to read, and (D) graph showing symptom change over the previous week.

The Interaktor app is generic and adjustable depending on the setting and situation. It is designed for both Android and iOS and can be downloaded to any smartphone or tablet and requires a separate log in. The primary features of the Interaktor app are regular assessment of self-reported symptoms, risk assessment models for alerts, continuous access to evidence-based self-care advice and links to relevant websites for more information, and graphs that allow patients to view their symptom reporting history (Figure 2).

The structure of the symptom assessment was inspired by a standardized symptom questionnaire that assesses a symptom’s occurrence, rated as “yes” or “no,” and a symptom’s frequency and distress level on a 4-point rating scale [22,23]. The pancreas version of Interaktor consists of 12 symptom questions following surgery and 3 additional questions for patients undergoing adjuvant chemotherapy, as defined by patients and health care professionals in our previous studies [19,20]. Patients also have the possibility to write a free-text comment before submitting a report. After completing the symptom assessment, the report is immediately sent to a secure server that is linked to a monitoring web interface where reports and alerts can be viewed. The risk assessment model for alerts is, in this version, programmed differently depending on whether patients undergo chemotherapy. There are two types of alerts: red and yellow. A red alert indicates that the patient is experiencing a severe symptom and should be contacted within 1 hour, and for yellow alerts, contact should be made the same day (Table 2). If an alert is triggered, the patient receives suggestions on self-care advice to read. Further, a text message is automatically sent to a cellphone at the clinic to notify the patient’s nurse to view the alerted symptoms in the web interface.

**Table 2:** Risk assessment model for alerts.

Symptom alerts		Rated as	Response options		Alert trigged after…		Type of alert
**After surgery**							
	Fever	Occurrence	“Yes”		One report		Red
	Pain	Frequency	“Almost always”		One report		Red
	Vomiting	Frequency	“Almost always”		One report		Red
	Dizziness	Frequency	“Almost always”		One report		Red
	Vomiting	Frequency	“Sometimes,” “Often,” OR “Almost always”		2 consecutive days		Yellow
	Dizziness	Frequency	“Sometimes,” “Often,” OR “Almost always”		2 consecutive days		Yellow
	Loose stool	Frequency	“Often” OR “Almost always”		3 consecutive days		Yellow
	Constipation	Occurrence	“Yes”		3 consecutive days		Yellow
	Eating difficulties	Frequency	“Often” OR “Almost always”		7 consecutive days		Yellow
	Pain	Frequency	“Often” OR “Almost always”		7 consecutive days		Yellow
	Nausea	Frequency	“Often” OR “Almost always”		7 consecutive days		Yellow
	Fatigue	Distress	“Rather much” OR “Very much”		7 consecutive days		Yellow
	Sadness/depression/worry	Distress	“Rather much” OR “Very much”		7 consecutive days		Yellow
	Problems performing activities at home	Distress	“Rather much” OR “Very much”		7 consecutive days		Yellow
	Problems performing activities outside home	Distress	“Rather much” OR “Very much”		7 consecutive days		Yellow
**During chemotherapy^a^**							
	Fever	Occurrence	“Yes”		One report		Red
	Breathing difficulties	Frequency	“Almost always”		One report		Red
	Nausea	Frequency	“Almost always”		One report		Red
	Vomiting	Frequency	“Almost always”		One report		Red
	Numbness/tingling in hands and/or feet	Frequency	“Almost always”		One report		Red
	Eating difficulties	Frequency	“Almost always”		One report		Red
	Swelling/pain/redness from SVP^b^/PICC^c^	Occurrence	“Yes”		One report		Yellow
	Loose stool	Frequency	“Almost always”		One report		Yellow
	Pain	Frequency	“Almost always”		One report		Yellow
	Dizziness	Frequency	“Almost always” OR “Often”		One report		Yellow
	Vomiting	Frequency	“Often”		One report		Yellow
	Nausea	Frequency	“Often”		One report		Yellow
	Breathing difficulties	Frequency	“Often”		One report		Yellow
	Constipation	Distress	“Very much”		One report		Yellow
	Sadness/depression/worry	Distress	“Very much”		One report		Yellow

^a^Since the patients undergoing chemotherapy have contact with a nurse at least once a week, no alerts were programmed to be triggered after multiple consecutive days.

^b^SVP: subcutaneous venous port.

^c^PICC: peripherally inserted central catheter.

### Procedure

A researcher helped the patients to download the app to their own smartphone. Patients who did not have access to a smartphone (n=2) were lent one with the app installed. The researcher instructed the patient on the different features; thereafter, the patient practiced submitting a report under the researcher’s supervision. The submitted report was then shown in the graphs and discussed together. The self-care advice, including hyperlinks to websites, was introduced. Furthermore, a written manual for using the app was given to the patients to take home. The patients were instructed to report symptoms daily for at least 4 weeks starting the first day after discharge from the surgical or rehabilitation clinic and up to 6 months after surgery or one week after ceasing adjuvant chemotherapy. After the first 4 weeks of reporting, a researcher called the patients to ask if they wanted to continue using the app. A reminder notification to report was sent through the app every day. The patients were thoroughly informed both orally and in writing that, in case of an alert, they would only be contacted during working hours (8 am to 4 pm on non-weekend days) because the report could only be monitored by a nurse during this time. If an alert was triggered outside of working hours, the patients were called the following weekday.

The patients’ contact nurses were responsible for monitoring alerts. They were employed at the surgical clinic or at the oncology clinic for those patients who underwent adjuvant chemotherapy. The nurses were instructed to call the patients if they received an alert text. One of the researchers could be contacted in the event of any technical problems. Patients who had access to advanced home care with specific home care nurses could not use the app since those nurses were not introduced to the app.

### Data Collection

Data concerning the number of submitted reports, reported symptoms, triggered alerts, and viewed self-care advice were logged on a secure server and extracted as an encrypted Excel file.

The patients were interviewed individually after their final report about their experiences with using the app. One patient died within the study period and therefore could not be interviewed. To ensure trustworthiness, the interviews followed a semistructured interview guide with the questions: “What was it like to use the app?” “In which way have you been in contact with health care?” and “In which way have you been able to be involved in your care?” Depending on the extent of the patients’ answers, probing questions like “Can you elaborate or give an example” were used. The interviews lasted for a median time of 31 minutes (range 16-71 minutes) and were audio recorded. To ensure that the patients were comfortable, they were interviewed either in their own home (n=21) or at the hospital (n=4) according to their own choice.

### Data Analysis

Logged data from the app were analyzed with descriptive statistics. Adherence to reporting was calculated as the number of days a patient submitted a report divided by the number of days a patient was meant to report and presented as a percentage.

The patients’ interviews were analyzed using thematic analysis, as described by Braun and Clark [24]. First, all interviews were transcribed verbatim and read through several times. Statements regarding the app were systematically coded throughout the entire dataset with an inductive approach. A code could consist of a few words or a whole sentence. Matching codes were then put together and created themes. All data in one theme were then reviewed to see if the theme worked in relation to the codes. This reviewing process was completed by all authors. If a theme did not work, the process of collating codes started from the beginning until all themes worked in relation to the codes and the entire dataset. During the whole process, themes were defined, named, and renamed. Individual quotes were chosen to validate the findings. To establish rigor of the analysis, the 15-point checklist of criteria by Braun and Clark [24] for good thematic analysis was followed [24].

## Results

### Logged Data

Patients used the app for a median of 190 days (range 35-245 days). The median adherence to reporting daily was 82.2% (range 23.5%-100%). Reasons to stop reporting in advance were own choice (n=1), follow-up care transferred to unit not included in the study (n=3), or death (n=1).

#### Reported Symptoms

A total of 6320 symptoms (median 170, range 9-994) were reported, and at the group level, all symptoms were reported but not by each patient (Table 3). The 4-point rating values were all used in the follow-up questions. Levels of frequency and distress of a symptom were mostly concordant except for nausea, vomiting, and dizziness, for which patients reported a higher distress level than frequency and the opposite for numbness in hands or feet (Table 3). Fatigue and pain were the most frequently occurring symptoms and also reported by most patients (Table 3).

**Table 3:** Occurrences, frequency, and distress of the symptoms as reported in the app by patients (n=26) following pancreaticoduodenectomy due to cancer.

Symptoms (number of patients reporting the symptom)	Occurrence (n=6320)			Frequency		Distress	
	n (%)	Median	Range	Mean (SD)	Range	Mean (SD)	Range
Fatigue (n=24)	1445 (22.86)	35.5	3-198	2.4 (0.47)	1-4	2.3 (0.42)	1-4
Pain (n=23)	863 (13.7)	19	1-169	2.1 (0.38)	1-4	2.3 (0.33)	1-4
Problems performing activities outside home (n=21)	605 (9.6)	21	1-161	2.4 (0.60)	1-4	2.3 (0.42)	1-4
Nausea (n=21)	572 (9.1)	11	1-158	1.9 (0.40)	1-4	2.3 (0.50)	1-4
Eating difficulties (n=22)	535 (8.5)	13.5	2-160	2.4 (0.62)	1-4	2.5 (0.48)	1-4
Loose stool (n=24)	526 (8.3)	6	1-133	2.1 (0.71)	1-4	2.1 (0.52)	1-4
Problems performing activities at home (n=20)	518 (8.2)	12.5	1-127	N/A^a^	N/A	2.3 (0.43)	1-4
Sadness, depression, worry (n=12)	386 (6.1)	14	3-169	2.3 (0.62)	1-4	2.3 (0.55)	1-4
Dizziness (n=15)	267 (4.2)	10	1-91	1.9 (0.51)	1-4	2.3 (0.37)	1-4
Numbness in hands or feet^b^ (n=9)	204 (3.3)	2	1-85	2.2 (0.68)	1-4	1.8 (0.31)	1-4
Constipation (n=23)	132 (2.1)	4	1-28	N/A	N/A	2.2 (0.68)	1-4
Fever (n=16)	87 (1)	3	1-18	N/A	N/A	N/A	N/A
Swelling/pain/redness from SVP^c^/PICC^b,d^ (n=9)	69 (1)	3	1-49	N/A	N/A	N/A	N/A
Breathing difficulties^b^ (n=7)	61 (1)	3	1-41	2.2 (0.36)	1-4	2.0 (0.50)	1-4
Vomiting (n=14)	50 (0.8)	2.5	1-11	1.4 (0.34)	1-4	2.5 (0.65)	1-4

^a^N/A: not applicable.

^b^Symptoms only reported during adjuvant chemotherapy.

^c^SVP: subcutaneous venous port.

^d^PICC: peripherally inserted central catheter.

#### Alerts

The total number of alerts was 512 (median 9, range 0-87), and almost all patients (n=24) reported an alert. Of these alerts, 35.5% (182/512) were severe (red). The most common alert was fever, which was also triggered by most patients (Table 4).

**Table 4:** Distribution of the number of alerts (n=512) reported in the app by patients (n=24) after discharge following pancreaticoduodenectomy due to cancer.

Symptom alerts (number of patients generating the alert)	Median (Range)	Red alerts (n=182), n	Yellow alerts (n=330), n
Fever (n=16)	3 (1-18)	87	N/A^a^
Dizziness (n=13)	5 (0-20)	0	67
PICC^b^ (n=9)	3 (1-51)	N/A	72
Loose stool (n=9)	2 (1-48)	N/A	71
Nausea (n=8)	2.5 (1-13)	1	34
Pain (n=8)	2.5 (1-5)	8	11
Eating difficulties (n=7)	2 (1-18)	5	9
Constipation (n=7)	1 (1-3)	N/A	10
Fatigue (n=6)	3 (1-4)	N/A	15
Problems with activities outside home (n=5)	4 (1-7)	N/A	19
Vomiting (n=5)	1 (1-1)	1	4
Breathing (n=4)	1.5 (1-6)	N/A	10
Problems with activities at home (n=3)	1 (1-1)	N/A	3
Sadness, depression, worry (n=2)	2.5 (2-3)	N/A	5
Numbness (n=1)	80 (80-80)	80	N/A

^a^N/A: not applicable.

^b^PICC: peripherally inserted central catheter.

#### Free-Text Comments

The free-text comment section to communicate with health care was used 302 times in total (median 7.5, range 0-90) and used by most patients (n=24). Most comments were a detailed description about a symptom, which was sometimes followed by a wish for counseling or the text “You do not need to call me.” The patients also used the free-text comment section to document values for weight, blood glucose, blood pressure, and temperature or to inform on admission to hospital, going away on holiday, or need for prescriptions.

#### Self-Care Advice

The patients viewed self-care advice 1231 times in total (median 30.5, range 3-181). The most commonly and least commonly viewed self-care advice is shown in Table 5.

**Table 5:** The five most and least commonly viewed self-care advice items and number of times viewed by the whole group

Self-care advice (number of patients who viewed the advice)		Number of times viewed
**Most commonly viewed**		
	Pancreatic enzyme supplement (n=25)	99
	Dietary advice (n=21)	86
	Pain (n=21)	76
	Fever (n=16)	68
	Weight loss (n=18)	62
**Least commonly viewed**		
	Sleep disturbance (n=6)	19
	Instable blood sugar (n=12)	19
	Breathing difficulties (n=5)	21
	Hair/skin/mucous membrane (n=5)	27
	Numbness/tingling in hands and feet (n=8)	27

### Interviews With Patients

**Figure 3:**
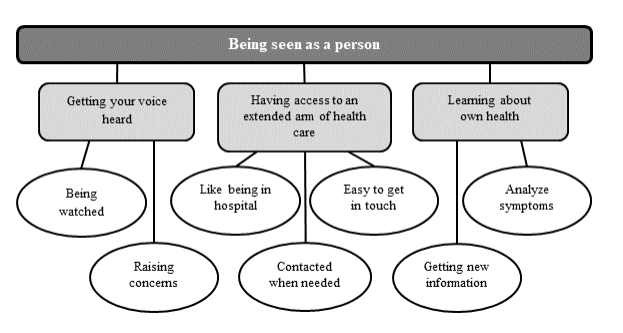
Examples of codes (white ovals) connected to the 3 sub-themes (light gray rectangles) and overarching theme (dark gray rectangle) identified through the thematic analysis of interviews with patients (n=25) using the app following pancreaticoduodenectomy due to cancer.

The overarching theme “Being seen as a person” was identified, with the following subthemes: “Getting your voice heard,” “Having access to an extended arm of health care,” and “Learning about own health.” Examples of codes connected to the subthemes are illustrated in Figure 3.

The overarching theme “Being seen as a person” reflects how the patients described how the app had supported them in being personally involved in their care and that care was based on their personal needs. Furthermore, they expressed that they felt secure and had a relationship with the health professionals. Despite care being delivered through an app, the patients expressed being seen as a person, a person beyond the disease.

#### Being Seen as a Person: Getting Your Voice Heard

The patients overall talked about feeling taken care of at home since someone was keeping an eye on how they were feeling on a daily basis. Patients undergoing chemotherapy noticed that the nurses had viewed their reports and knew how they were feeling when they came to the hospital.

You just have to send in your report and then you get to talk to someone. /…/ I think you are more involved in care this way since you have your voice heard when you want.

Patient 8

When the patients had reported an alerting symptom, they expressed how important it was that the nurse called the patient. This conversation with the nurse resulted in individually adjusted advice. The patients felt that they received comfort and help with their problems from the nurses who called after an alert and that they could raise issues other than the reported symptoms.

Once I could not understand a manual I was reading, my brain wasn’t working, and then I got… afraid. Then I wrote that in the free text and the nurse called and told me about ‘cyto-brain’. It was comforting to get an explanation and to talk about it instead of going around worrying about it alone.

Patient 5

#### Being Seen as a Person: Having Access to an Extended Arm of Health Care

The patients described the app as a reassuring link to health care after discharge and that it made them feel like they were still at the hospital even if they were at home. Only having one point of contact and not having to think about who to call was described as a benefit. Patients expressed that they often knew the nurse who contacted them or learned to recognize the voice of nurses they had not met in person. Sometimes the patients did not know or recognize the nurse but the person who called was described as always being well informed. The patients expressed that the app offered a faster and easier way to get in touch with health care than the regular way, which is to call, enter contact information, and either be placed on hold or called back later. The need for fast and easy contact with health care was most prominent during the first weeks when a lot of symptoms were present but also later if new symptoms arose due to chemotherapy.

Someone is checking up on you, so that you are not starting to feel too bad. And that is great. It’s something in-between being at home and lying in hospital, but at home you are free.

Patient 12

Using the app was a sort of follow-up that was otherwise lacking, and more contact with health care was initiated since they would not themselves have called as often if the decision was theirs. Sometimes the patients were not contacted after an alert since the report was submitted outside working hours. This resulted in an empty feeling, as it is during weekends and nights that feelings of loneliness can arise and thereby the need to talk to someone. A wish that contact should always be made when needed was raised. However, other patients expressed no problems with this and said that in case of serious problems, they would have contacted health care themselves. Some patients wanted to decide by themselves if a nurse should contact them since they sometimes had been contacted when they did not have any need for contact. Patients who felt no need to be called learned to adjust their responses so that an alert would not be triggered or used the free text to write a message to the nurse.

The app decides when you will be contacted and that feels a bit weird, because sometimes it's okay, but sometimes it's not okay, and then the nurse and I agreed that when I reported symptoms and didn’t want, or need, to be contacted, I wrote that in the free text.

Patient 16

Other patients found it reassuring that someone else was responsible for making the decision if contact was needed and knowing that if someone did not call, everything was satisfactory.

The app made me not have to judge myself what is cause for concern. Instead I could leave that to someone else. Not having to think about if it was something I needed to react to, but instead just hand myself over.

Patient 17

#### Being Seen as a Person: Learning About Own Health

The patients could identify important symptoms and reflect on how they felt since they were asked to rate symptoms daily.

To think about how you feel every day is a perspective that I think is especially beneficial, because it is very easy to think that you are completely well and then you push yourself too much.

Patient 4

Some patients thought it was helpful to analyze their symptom change over time when symptoms had been unstable.

I was curious to see if my symptoms, like lack of appetite and tiredness, were connected to the treatment. And it seems that the day after treatment, and the following two or three days, then the tiredness is at its worst, whereas changes in appetite are much slower.

Patient 5

Having access to self-care advice provided new knowledge on symptoms and how to manage them and gave explanations as to why they were feeling as they were, and misconceptions could be dealt with.

I think reading the advice has been valuable to be able to justify, why it's true, why I feel like I do, or if there is something I need to think about.

Patient 18

The patients expressed that having easy access to the advice was important since information is easy to forget, they might not have been given enough information before discharge, or they were not able to absorb information at that time.

I understand that the staff don’t have time to explain everything, or that you are not in the right frame of mind to understand everything they tell you. It was good to have the app directly after being discharged following the surgery and at that time I used that self-care feature a lot /…/ when you Google you can end up on strange sites that don’t reflect your situation so this was more straightforward and concise and contains 100% facts.

Patient 5

## Discussion

### Principal Findings

This study shows that using an interactive app for symptom reporting and management is accepted by patients who have undergone pancreaticoduodenectomy due to cancer and enables person-centered care after discharge. The findings confirm the intent of Interaktor to offer a support system that provides several features that address individual supportive care needs. Our previous results have shown that patients who used the app experienced higher emotional function, less symptom burden, and higher self-care activity levels after surgery compared to patients not using the app [21], which is supported by the results in the present study. There was large variation in how patients used the app and interacted with the nurses, for example, how they wrote free-text comments and viewed self-care advice. Irrespective of how the patients used the app’s features, their experiences with using it were similar. The patients described how the app gave them reassurance in being monitored and having contact with health care, as well as receiving support for self-care.

### Limitations

Although 115 patients were approached before surgery, data from only 26 patients could be analyzed. Many patients were not eligible upon discharge due to the severity of the disease or treatment, showing the complexity with including this patient group in clinical trials. The initial consent rate was high, specifically 69.6% (80 consented of 115 approached), a rate comparable to a feasibility study of a similar intervention [25]. The consent rate might have been even higher if patients were approached upon discharge when they are more focused on their need for supportive care at home. Patients who declined to participate in the study may have been less interested or experienced obstacles in using a smartphone app compared to those patients who consented. Interest and ability to use mHealth are likely to constantly grow as smartphone access is increasing every year. For instance, recent mapping shows that 90% of the Swedish population have access to a smartphone [26]. Some patients brought up that they had forgotten about some of the features of the app that must be considered when interpreting the results. In future studies of Interaktor and other mHealth tools, it is advisable to make time for a number of training opportunities. In this study, monitoring of and response to alerts could only be made during working hours on weekdays due to the organizational structure at the participating clinics. In future studies, and especially if the app should be implemented in standard care, monitoring of and response to alerts should be made at all hours of the day and not just restricted to certain hours.

### Comparison to Prior Work

The patients had a median adherence of 82.2% for reporting symptoms, which can be considered as high, especially since the reporting period was 6 months. Some patients even reached 100% adherence, meaning that they reported symptoms every day for 6 months. This is a major strength of this study and shows the participating patients’ interest and need to use the app. The high median adherence rate has been shown in patients with prostate cancer using Interaktor during radiotherapy treatment [27]. All assessed symptoms were reported in the app, and the patients perceived that the questions covered all experienced symptoms and that specifications could be made in the free text if needed. Interestingly, the patients’ responses on the 4-point rating scale of a symptom’s frequency and distress level were concordant for most symptoms. The coherent responses indicate that it is enough to ask for symptom occurrence, rated by “yes” or “no,” and then either frequency or distress, an approach previously evaluated to be sufficient [28]. Not only do the findings provide knowledge about which symptoms patients normally experience following pancreaticoduodenectomy, they also show that there is large individual spread between symptom experiences. Likewise, there was large spread in how many alerts the patients triggered. Of the reported symptoms, 8% triggered an alert. Even so, none of the patients in the present study expressed that alerts were triggered too seldom. On the contrary, a few patients felt that alerts had been triggered when they felt no need to be contacted. They had then learnt to adjust their responses so that an alert would not be triggered or used the free text to communicate if they did not want to be contacted, a strategy also described by patients with prostate cancer [27]. The possibility to write a free-text message was highly used and appreciated, not only to communicate whether contact was needed but also to raise other needs. Based on the results, the risk assessment model seems adequate for patients with pancreatic cancer with the added possibility to write a free-text message. At a group level, all self-care advice included in the app was viewed, although there was large variety in how often patients viewed the advice. The findings show a pattern where the most occurring symptoms are linked to the most viewed self-care advice. This shows the importance of having advice connected to experienced symptoms and that the app targets individual needs.

Person-centered care is defined as shifting the focus from the disease to the person with the illness — a person with individual needs and preferences — and by doing so, the person can be engaged as an active partner in his or her own care and treatment [9]. The results show several ways in which the app facilitates person-centered care by targeting individual needs, namely, by viewing self-care advice as often as needed and connected to experienced symptoms, communicating to the nurse through free-text messages, analyzing one’s own symptoms, getting individual advice following an alert and call by the nurse, and experiencing an easier way to contact health care. By targeting these needs, the contact and care after discharge can be tailored to the patient’s needs and preferences and not according to a standardized disease-specific schedule.

The findings that the patients got support for symptom management and felt reassured in being monitored and having an easy way to stay in contact with health care are consistent with experiences from patients with other types of cancer [27,29,30]. Moreover, being monitored and contacted after submitting a report has been experienced as participating in one’s own care by patients with colorectal cancer who used a cellphone-based system to report side effects during chemotherapy treatment to health care providers [31] and by patients with prostate cancer using Interaktor to report side effects during radiotherapy [32]. In this study, patients’ participation was also evident when patients made agreements with their nurse as to when they needed to be contacted or adjusted their responses when they did not want to be contacted. Also, patients created relationships with the nurses and shared knowledge and information in connection with an alert and increased their own knowledge by viewing self-care advice, aspects determined to be vital for patient participation in previous studies [33]. It has been stated that illness and poor health could hinder patient participation [33]. However, in the interviews, patients described that the need to use the app was most relevant during times when they felt most unwell. As such, using an app like Interaktor can support patients with poor health to enhance their wellbeing and participate in their own care.

Most discrepancies in opinions about the app concerned the text message that was automatically sent to a nurse if an alert was triggered. Some patients wanted to decide for themselves whether to be contacted while others thought that it was reassuring to know that the decision was somebody else’s. Considering previous results showing that cancer survivors feel unable to judge the seriousness of their symptoms [34], it does not seem to be wise to lay the full responsibility for contact with health care on the unwell patient. However, in further adjustments of the app, these opinions need to be addressed, for instance by offering patients an easy way to communicate whether they wish to be contacted and the reason why.

Patients in this study did not feel there were any negative aspects in answering questions about symptoms. On the contrary, it was found helpful to identify important symptoms and reflect on how they felt in a rational and conscious manner. Similar positive statements have been made by patients with prostate cancer using Interaktor [27]. However, these experiences are in contrast to patients using another self-reporting cellphone-based system where answering questions about side effects of treatment sometimes made patients aware of their side effects in a negative way, causing upsetting emotions [31]. The discrepancies could be due to patients in the latter study not being able to view self-care advice in connection with their reported symptoms.

### Conclusion

The Interaktor app proved to be well accepted by patients following pancreaticoduodenectomy due to cancer. It made patients feel reassured at home and offered support for self-care. Also, the app facilitated person-centered care through its multiple features targeting individual supportive care needs and enabled participation in own care. This supports our recent studies showing that patients using the app had less symptom burden and higher self-care activity levels than those only getting standard care. This study shows that there are good reasons to implement mHealth support systems for patients with pancreatic cancer.

## Abbreviations

mHealth: mobile health.
N/A: not applicable.
PICC: peripherally inserted central catheter.
SVP: subcutaneous venous port.
